# Distinct molecular subtypes of papillary thyroid carcinoma and gene signature with diagnostic capability

**DOI:** 10.1038/s41388-022-02499-0

**Published:** 2022-10-17

**Authors:** Shubin Hong, Yubin Xie, Zhen Cheng, Jie Li, Weiman He, Zhuming Guo, Quan Zhang, Sui Peng, Minghui He, Shuang Yu, Lixia Xu, Rengyun Liu, Tianyi Xu, Yunjian Zhang, Yanbing Li, Jiguang Wang, Weiming Lv, Jun Yu, Haipeng Xiao

**Affiliations:** 1 Department of endocrinology, The First Affiliated Hospital of Sun Yat-sen University, Guangzhou, China; 2 Institute of Precision Medicine, The First Affiliated Hospital of Sun Yat-sen University, Guangzhou, China; 3 Department of Breast and Thyroid Surgery, The First Affiliated Hospital of Sun Yat-sen University, Guangzhou, China; 4 Department of Head and Neck Surgery, Sun Yat-sen University Cancer Center, Guangzhou, China; 5 Clinical Trials Unit, The First Affiliated Hospital of Sun Yat-sen University, Guangzhou, China; 6 Department of Oncology, The First Affiliated Hospital of Sun Yat-sen University, Guangzhou, China; 7 Division of Life Science, Department of Chemical and Biological Engineering, State Key Laboratory of Molecular Neuroscience and Hong Kong Center for Neurodegenerative Diseases, The Hong Kong University of Science and Technology, Hong Kong SAR, China; 8 Institute of Digestive Disease and Department of Medicine & Therapeutics, State Key Laboratory of Digestive Disease, The Chinese University of Hong Kong, Hong Kong SAR, China

**Keywords:** Cancer genomics, Thyroid cancer

## Abstract

Papillary thyroid carcinoma (PTC) is heterogeneous and its molecular characteristics remain elusive. We integrated transcriptomic sequencing, genomic analysis and clinicopathologic information from 582 tissue samples of 216 PTC and 75 benign thyroid nodule (BTN) patients. We discovered four subtypes of PTC including Immune-enriched Subtype, BRAF-enriched Subtype, Stromal Subtype and CNV-enriched Subtype. Molecular subtypes were validated in an external cohort of 497 PTC cases from the TCGA. Tumors in the Immune-enriched Subtype showed higher immune infiltration and overexpression of immune checkpoints, whilst BRAF-enriched Subtype showed a higher tendency for extrathyroidal extension and more advanced TNM stage. Key oncogenes including LRRK2, SLC34A2, MUC1, FOXQ1 and KRT19 were overexpressed and enriched in oncogenic MAPK and PI3K/AKT signaling pathways in BRAF-enriched subtype. Further analysis of BRAF-enriched Subtype identified three subclasses with different degrees of malignancies. We also uncovered the molecular link of the initiation and progression from BTN to subtypes of PTC using trajectory analysis. Moreover, a 20-gene expression signature was generated for differential diagnosis of PTC from BTN patients. Together, our work identified previously unreported molecular subtypes of PTC, offering opportunities to stratify patients into optimal treatment plans based on molecular subtyping.

## Introduction

Thyroid cancer, as the most common endocrine cancer, ranks the ninth most common malignant tumor in the world [1], with an annual growth rate of global incidence up to 20% [2]. As the most frequent pathological types, papillary thyroid cancer (PTC) accounts for more than 85% of the thyroid cancer [3]. Usually, PTC has a good prognosis with a 5-year survival rate of more than 90% after surgery, even some of them are considered unnecessary for immediate surgery. However, due to the multifocality and early lymph node metastasis of PTC, its recurrence rate is as high as 35% and the 10-year disease-specific survival rate of advance PTC is less than 50% [4, 5]. At present, TNM staging and recurrence risk stratification based on postoperative pathology are still the major approaches for evaluating the prognosis of PTC. The molecular characteristics of PTC with different biological features are barely known. Therefore, exploring the accurate and effective molecular classification of PTC patients will provide new insights for the prognosis assessment and individualized treatment for PTC patients.

Efforts have been taken to the molecular classification of PTC. The Cancer Genome Atlas (TCGA) project was the first pan-genomic study of thyroid cancer. The recent molecular classifications are based on the most frequent genetic events in PTC (BRAF mutation and RAS mutation) [6, 7]. However, the existing subtyping of thyroid cancer revolves around BRAF mutations and RAS mutations, which could not fully cover the molecular and clinical characteristics of PTC, without considering other genomic changes and the tumor microenvironment. Therefore, a comprehensive analysis that includes PTC patients with varied biological characteristics and combines with the expression profile of benign thyroid nodules (BTN) is needed to produce a model that can be highly correlated with the prognosis and enhance the understanding of the molecular mechanism of the disease.

In this study, we performed a comprehensive RNA sequencing (RNA-seq) in 291 paired tumor and non-tumor tissue samples (216 PTC patients and 75 BTN patients), to reveal their transcriptomic and genomic characteristics. Using consensus non-negative matrix factorization (NMF), we derived and refined 4 novel molecular subtypes in PTC and further validated their reproducibility in TCGA database. We showed that each of the 4 subtypes was associated with distinct genetic and transcriptomic characteristics, immune patterns and clinicopathological features. Besides, we screened out marker genes that can distinguish benign and malignant thyroid nodules. These findings will be expected to help optimize the diagnosis and treatment of thyroid cancer.

## Results

### Landscape of transcriptional and genomic alterations in thyroid nodules

**Fig. 1:**
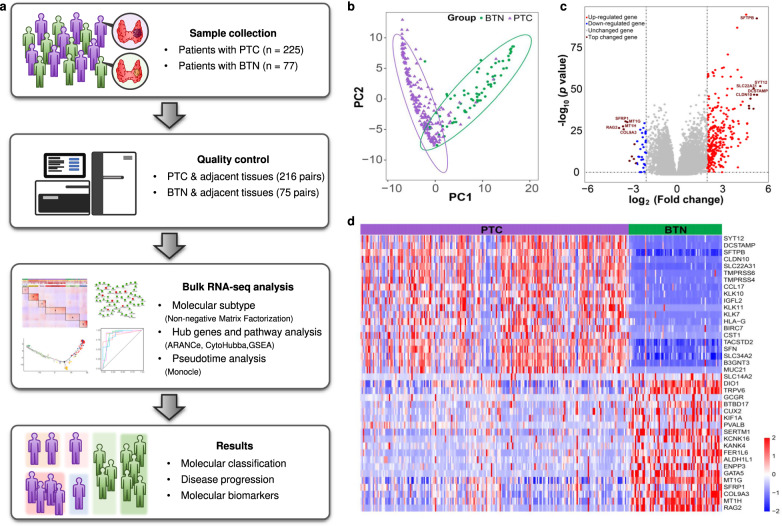
Landscape of transcriptional and genomic alterations in thyroid nodules. **a** Flow chart of the study. There were totally 291 patients with thyroid nodules in our study, including 216 PTC and 75 BTN. **b** Principal component analysis of all thyroid nodules. The type of nodules represented by color. **c** Volcano map of differentially expressed genes for BTN vs PTC. Red dots stand for up-regulated genes, blue dots stand for down-regulated genes, crimson dots stand for top changed genes, and grey dots stand for non-significantly changed genes according to the cut off *P* < 0.05 or log_2_(fold change) >1. **d** The heatmap of top 20 DEGs between BTN and PTC according to *P* < 0.05. PTC papillary thyroid cancer, BTN benign thyroid nodule.

To find the molecular characteristics of benign and malignant thyroid nodules, we conducted a comprehensive bulk RNA-seq analysis of 225 PTC and 77 BTN patients (Fig. 1a). After quality control of RNA sequencing, a total of 216 pairs of PTC and adjacent tissues and 75 pairs of BTN and adjacent tissues were finally admitted for the following analysis. As shown in principle component analysis (Fig. 1b), there was significant separation between BTN and PTC. A total of 3944 genes had their expression levels changed between BTN and PTC (Fig. 1c). Of which, the top 20 differential expressed genes (DEGs) were mainly related to the progression of malignant tumors, including SYT12, CLDN10, COL9A3, SFRP1, MT1G, MTIH, etc. (Fig. 1d).

Besides, we analyzed the gene mutation, gene fusion and copy number variation (CNV) of all samples based on the gene expression profiles (Supplementary Fig. 1a, b). BRAF mutation, account for 44.91% of PTC, was the main mutation in PTC, and almost all the BRAF mutations were T1799A substitutions (BRAF^V600E^). Since it is difficult to infer the arm-level variations of all chromosome by gene expression profiles, we used the CaSpER method to calculate the copy number variation (CNV) at the gene level, and identified significant losses or gains of 50 genes in 18.4% of PTC cases (Supplementary Fig. 1c). Through the gene fusion analysis, RET fusions were the most frequent type, accounting for 10.19% of PTC (Supplementary Fig. 1d). Among them, we found a novel RET fusion that retained the kinase domain, the KCTD5-RET gene fusion, and which was also verified by RT-PCR and sanger sequencing (Supplementary Fig. 1e, f).

### Transcriptome-based molecular classification in thyroid nodules

**Fig. 2:**
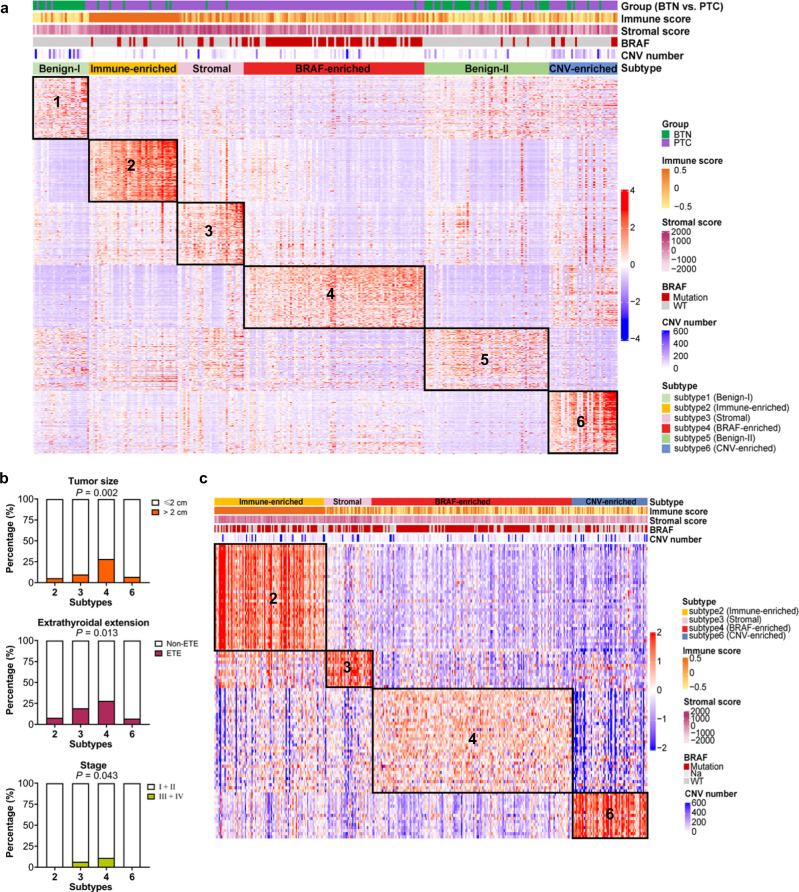
Molecular classification of thyroid nodules. **a** Transcriptome-based unsupervised classification of PTC using non-negative matrix factorization (NMF) consensus identified 6 subtypes of thyroid nodules: Benign-I, Immune-enriched, Stromal, BRAF-enriched, Benign-II, CNV-enriched subtypes. **b** Box plots representing the proportion of tumors greater than 2 cm in diameter, lymph node metastasis, and stage III/IV tumors in each subtype. **c** Heatmap showing molecular PTC subtypes prediction in TCGA cohort using a predefined gene classifier generated by NMF.

By performing unsupervised NMF clustering of 291 samples of benign and malignant thyroid nodule, we identified 6 subtypes, four of which dominated by PTC and two of which were mainly BTN (Fig. 2a). Further analysis of the characteristics of the 4 subtypes of PTC (Subtype 2, 3, 4 and 6) found that Subtype 2 had the highest immune score, so-called Immune-enriched Subtype, while Subtype 3 with the highest stromal score was called Stromal Subtype. BRAF^V600E^ mutations were mainly enriched in Subtype 4 (BRAF-enriched Subtype). Meanwhile, the proportion of CNV in Subtype 6 (CNV-enriched Subtype) was significantly higher than that of other subtypes (Fig. 2a). We then performed the clinicopathological data analysis in these 4 subtypes, and observed that PTC in BRAF-enriched Subtype were more prone to extrathyroidal extension than others. Besides, the majority of stage III/IV PTC were also clustered in BRAF-enriched Subtype (Fig. 2b and Supplementary Table 1). In terms of tumor multifocality, lymph node metastasis and distant metastasis, there were no statistically significant differences among these 4 subtypes.

In order to externally validate the molecular classification of PTC described, we constructed a molecular classifier composed of a set of marker genes of each subtype, and verified its specificity for each subtype through the random forest method. The 497 PTC cases in TCGA cohort were allocated into 4 subtypes by using this molecular classifier (Fig. 2c). The characteristics of each subtype was consistent, implying the robustness of our molecular classification system.

To identify the potential hub genes of the molecular characteristics in each PTC subtypes, the ARACNe and GSEA methods were used to construct a gene-pathway co-expression network. In the subtypes dominated by BTN (Subtype 1 and 5), amino acid metabolism and lipid metabolism pathways were up-regulated while the MAPK pathway, the JAK-STAT pathway, and various immune cell functions were downregulated, including T cells, B cells and NK cells (Supplementary Fig. 2a, b). Among the 4 subtypes of PTC, the hub genes of the Stromal Subtype mainly encode extracellular matrix proteins, which were involved in regulating a variety of classic oncogenic pathway, including the MAPK signaling, the Wnt signaling, the TGF-beta signaling pathway (Supplementary Fig. 3a, c). In CNV-enriched Subtype, most of the hub genes, including TMSB10, were oncogenes, while several oncogenic pathways and immune pathways were down-regulated (Supplementary Fig. 3b, d).

### Characteristics of Immune-enriched Subtype

**Fig. 3:**
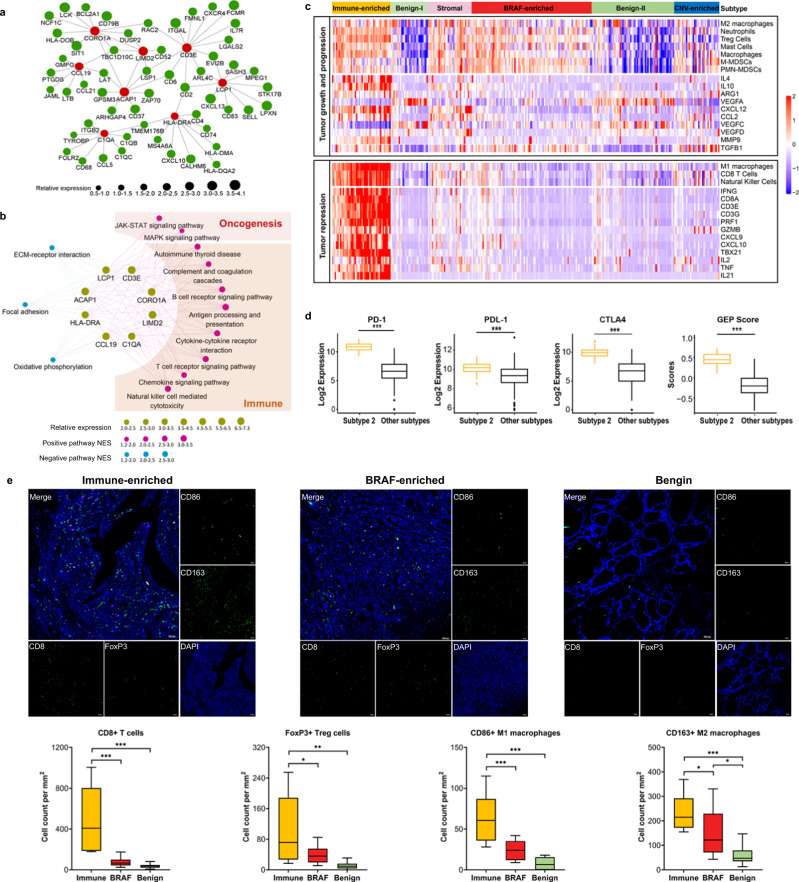
Characteristics of Immune-enriched Subtype. **a** Gene correlation network represents the hub genes of Immune-enriched Subtype identified by ARACNe method. Red dots stand for the top hub genes in the network. The dot’s size represents the relative expression level of each gene. **b** The gene-pathway co-expression network of Immune-enriched Subtype by the ARACNe and GSEA method. Dots in the center of the network stand for top hub genes, and the size of dots represents the relative expression level of each gene. Red dots and blue dots in the outer circle stand for the downstream upregulated and down regulated pathways of the hub genes, and dots’ size represents normalized enrichment score (NES). **c** Heatmap represents enrichment scores for immune signatures and gene expression levels in the Immune-enriched Subtype and other subtypes. **d** Box plots representing the relative expression of PD-1, PDL-1, CTLA4 and T cell inflamed GEP score in Immune-enriched Subtype and other subtypes. **e** The mIHC showed that several immune cells infiltration (CD8 + T cell, FoxP3+ Treg cells, CD86 + M1 macrophage and CD163 + M2 macrophage) in PTC tissues, BTN tissues and adjacent normal tissues. NES, normalized enrichment score; GEP, gene expression profile; mIHC, multiplexed immunohistochemistry.

We found that most of the key regulated genes of Immune-enriched Subtype were related to the function of immune cells, including CORO1A, CD3E, LCP1, ACAP1, HLA-DRA, CCL19 and RAC2 (Fig. 3a). Similarly, gene set enrichment analysis showed enrichment of antigen processing and presentation, T cell differentiation (Th17, Th1 and Th2 differentiation), autoimmune thyroid disease, the NF-κB signaling, chemokine and cytokine signaling. Besides, two classical oncogenic pathways, the JAK-STAT and the MAPK pathway were also activated in Immune-enriched Subtype (Fig. 3b). We conducted immunophenotyping to gain further biological insight into the immunologic nature of the Immune-enriched Subtype (Fig. 3c). Patients belonging to Immune-enriched, Stromal, and BRAF-enriched subtypes showed enrichment of immunosuppressive components, such as Treg, M2 macrophages, M-MDSCs and TGF-β signaling. But Immune-enriched Subtype was also associated with active immune response, including enrichment of antitumor immune cells (eg, CD8 T cells, M1 macrophages, NK cells) and overexpression of adaptive immune response genes (eg, CD8A, TNF, GZMB, PRF1, IFNG). Besides, the expression of PD-1, PD-L1, CTLA4 and the T cell inflamed GEP score in Immune-enriched Subtype were higher than other subtypes (Fig. 3d). The immune infiltration status among subtypes were further confirmed by mIHC, which showed that the infiltration of CD8 + T cell, FoxP3+ Treg cells, CD86 + M1 macrophages and CD163 + M2 macrophages were significantly higher in Immune-enriched Subtype than in BRAF-enriched Subtype and BTN Subtype (Fig. 3e).

### Characteristics of BRAF-enriched Subtype

**Fig. 4:**
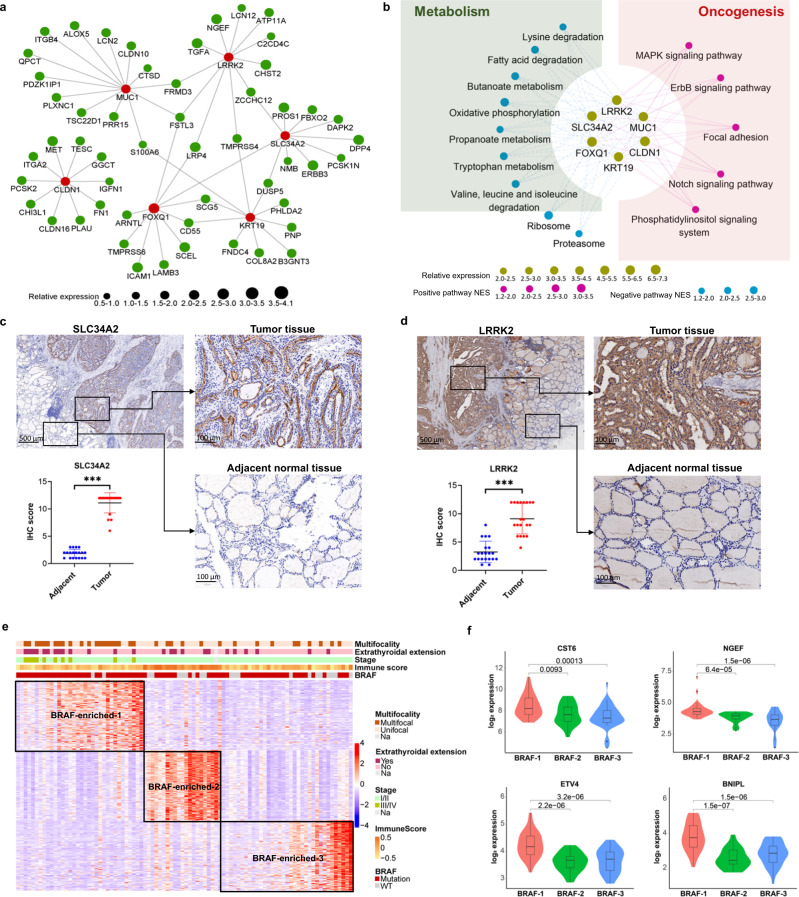
Characteristics of BRAF-enriched Subtype. **a** Gene correlation network represents the hub genes of BRAF-enriched Subtype identified by ARACNe method. Red dots stand for the top hub genes in the network. The dot’s size represents the relative expression level of each gene. **b** The gene-pathway co-expression network of BRAF-enriched Subtype by the ARACNe and GSEA method. Dots in the center of the network stand for top hub genes, and the size of dots represents the relative expression level of each gene. Red dots and blue dots in the outer circle stand for the downstream upregulated and down regulated pathways of the hub genes, and dots’ size represents NES. **c** The expression of SLC34A2 and **d** LRRK2 in PTC tissues and adjacent thyroid tissues of patients belong to BRAF-enriched subtype. **e** Further classification analysis of BRAF-enriched Subtype using NMF. **f** Box plots representing the relative expression of hub genes of BRAF-enriched-1 subgroup, including CST6, NGEF, ETV4 and BNIPL.

To further analyze the distribution of genetic alterations in PTC subtypes, we found that a majority of BRAF mutation (75%) and RET gene fusions were clustered in Subtype 4 (BRAF-enriched Subtype). Several hub genes of BRAF-enriched Subtype were closely related to BRAF^V600E^ mutations, including MUC1, LRRK2, SLC34A2, FOXQ1 and KRT19 (Fig. 4a). Gene set enrichment analysis identified the classical BRAF mutation related signaling, the MAPK signaling (Fig. 4b). In addition, the pathways related to tumor metastasis were also enriched in BRAF-enriched Subtype, including the Notch signaling and focal adhesion (Fig. 4b).

The expression levels and functions of the key regulatory genes of BRAF-enriched subtype were explored using IHC and cell function experiments. It is shown that both LRRK2 and SLC34A2 were highly expressed in BRAF-enriched subtype (Fig. 4c, d). The effect of LRRK2 on thyroid cancer progression was determined by series in vivo and in vitro experiments. Two thyroid cancer cell lines, BCPAP and KHM-5M were transfected with siRNA targeting LRRK2, and the knockdown efficiency were confirmed by qPCR and Western blot (Supplementary Fig. 4a, b). Knockdown of LRRK2 significantly suppressed the growth of thyroid cancer cell in vitro and in vivo (Supplementary Fig. 4c–f).

PTC patients with BRAF mutation have various clinicopathological features and clinical outcomes [8]. We conducted a sub-classification analysis of BRAF-enriched Subtype and identified three subclasses (Fig. 4e). The subclass 1 (BRAF-enriched-1) had the highest proportion of extrathyroidal invasion (Fig. 4e). We further searched whether there are abnormal expressions of certain genes causing such clinical changes, and found that several oncogenes were significantly upregulated in BRAF-enriched-1 subtype, including CST6, NGEF, ETV4 and BNIPL, compared to BRAF-enriched 2 and BRAF-enriched 3 subtypes (Fig. 4f).

### Gene expression landscape of benign and malignant thyroid nodules

**Fig. 5:**
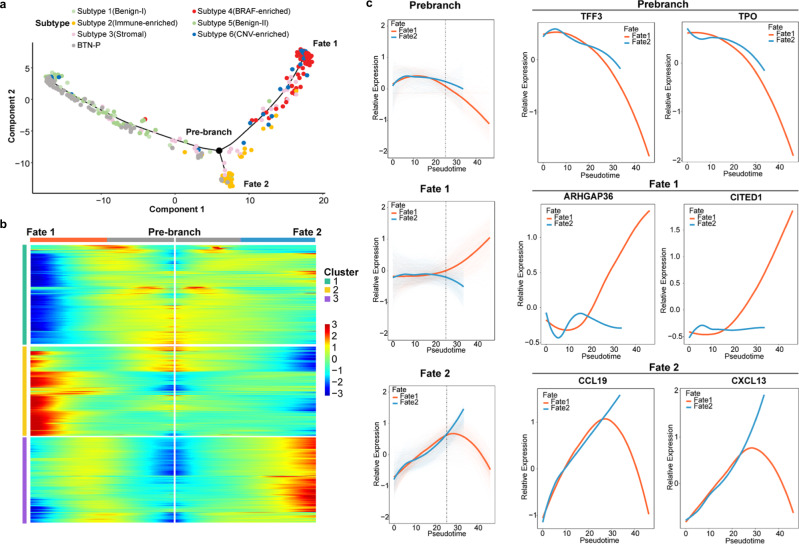
Gene expression dynamics of thyroid nodules. **a** The distribution of subtypes on the pseudotime trajectory constructed according to the transcriptional profiles. Each point represents a patient with colors corresponding to the subtypes it belongs to. **b** Gene expression heatmap of 2582 top DEGs (cataloged in three clusters) in a pseudo-temporal order with pre-branch shown on the middle, upper branch (Fate 1) and lower branch (Fate 2) shown on the right and left respectively. **c** The expression dynamics of 2582 top DEGs were cataloged into three clusters in a pseudotime manner shown as red lines (Fate 1) and blue lines (Fate 2). Thick lines indicate the average gene expression level in each cluster. The expression patterns of representative genes in each gene cluster were shown on the right.

The gene expression profiling data of all the thyroid nodules allowed us to construct the thyroid nodules trajectory to further investigate the heterogeneity and the potential transition from BTN to PTC. The pseudotime trajectory axis derived from Monocle indicated that the BTNs were closed to BTN-adjacent thyroid tissues, which were mostly located at the beginning of the trajectory (Fig. 5a). Then, they transdifferentiated into PTCs, and PTCs bifurcated into two diverse branches in the latter part of the trajectory (Fig. 5a and Supplementary Fig. 5a). By mapping the TDS in the trajectory, we found that the decreased differentiation degree of thyroid nodules along this trajectory (Supplementary Fig. 5b). As we found that malignant nodules are divided into two branches in the pseudo-chronological trajectory, we further analyzed the distribution of four PTC subtypes in the trajectory (Fig. 5a). The immune score increased along the trajectory and reached the highest at the end of the lower branch, where the Immune-enriched Subtype was mainly distributed (Supplementary Fig. 5c). Besides, the BRAF-enriched Subtype was mainly distributed in the upper branch with lowest TDS (Supplementary Fig. 5d).

To gain insights into the gene expression dynamics along the trajectory, we analyzed the expression changes of 2582 top differential expression genes and observed three gene clusters in characterized patterns (Fig. 5b). Genes in cluster I (pre-branch) were largely involved in the biological process of thyroid gland (e.g., TFF3 and TPO) and were gradually downregulated along the trajectory (Fig. 5c). Cluster II genes were activated at the end of the upper branch, and most of them were reported to promote PTC progression (e.g., ARHGAP and CITED1) (Fig. 5c). Finally, the cluster III genes were mostly related to immune reaction (e.g., CCL19 and CXCL13) and were upregulated and maintained at high expression levels until the final stage of lower branch (Fig. 5c). The distribution of four PTC subtypes on the trajectory were also confirmed by pathway enrichment analysis, as the lower branch genes were mainly enriched in immune pathways and the upper branch genes displayed an enrichment of classical oncogenic pathways (Supplementary Fig. 5e).

### A gene signature for the differentiation of PTC from BTN

**Fig. 6:**
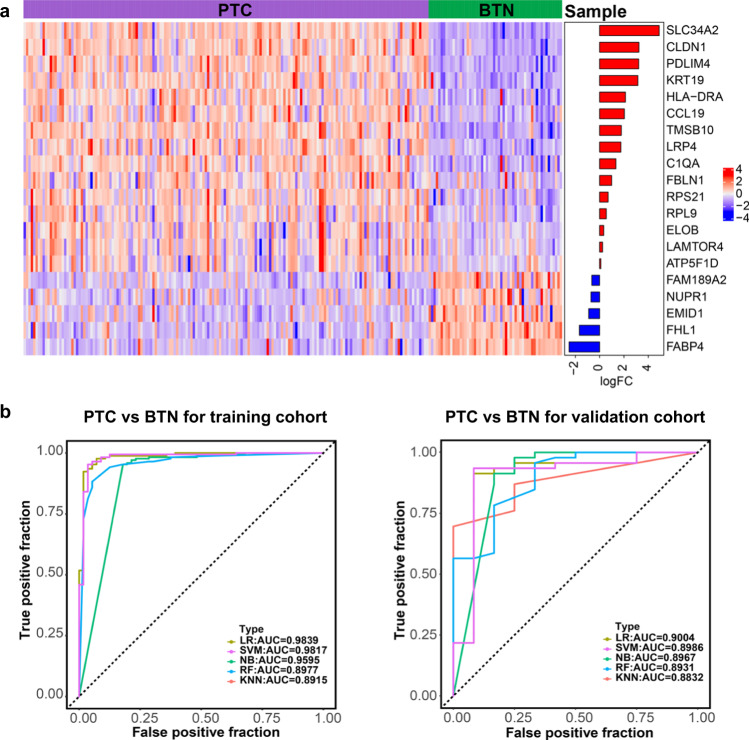
An integrated diagnosis model for PTC. **a** The heatmap of 20 DEGs identified by recursive feature elimination and logistic regression analysis for discriminating PTC and BTN. **b** Receiver operating characteristic (ROC) analysis for discriminating PTC from BTN based on Logistic Regression (LR), Support Vector Machine (SVM), Naïve Bayes (NB), Random Forest (RF) and K-Nearest Neighbor (KNN) algorithm in training cohort (left) and validation cohort (right).

We next sought to derive gene signatures that could be used to improve the diagnosis and prognosis assessment of PTC. We randomly divided all samples into training cohort and validation cohort according to the ratio of 8:2 and ensured consistent negative and positive ratios in both cohorts. We used recursive feature elimination and logistic regression analysis to derive a 20-gene expression signature (Fig. 6a), and the AUC of the ROC test is 0.9839 (Fig. 6b left). To validate the utility of the gene expression signature, we tested the 20-gene panel in independent validation cohort and the AUC is 0.9004 (Fig. 6b right), which is a set of excellent biomarkers for the differentiating PTC from BTN.

## Discussion

Thyroid cancer lacks a mature and practical molecular classification system like other cancers do [6, 7, 9]. To bridge this knowledge gap, we provided an integrative transcriptome and genomic landscape of a large Chinese cohort of PTC and BTN, as well as key clinical traits. By comprehensively analyzing the transcriptome profile of PTC and BTN, 4 molecular subtypes (Immune-enriched, Stromal, BRAF-enriched and CNV-enriched) with identical biological characteristics and driver genes were identified in our cohort and confirmed in TCGA cohort. We found that the pathological characteristics of thyroid carcinomas including extrathyroidal extension and TNM stage were associated with this molecular subtyping system. Furthermore, a 20-gene expression signature was generated for diagnosis of PTC from BTN, which can be potentially apply in the clinical practice in the future by less costive qPCR. These data provided a comprehensive elaboration of the molecular portrait of PTC and pave the way for more accurate disease evaluation and treatment.

It is well established that the two most common mutations, BRAF and RAS mutations, play an important role in the molecular subtyping of PTC [10, 11]. A portion of samples with different molecular characteristics from the former two subtypes, were classified into a new subtype, named Non-BRAF-Non-RAS subtype [7]. However, gene mutation could not fully explain the molecular features of PTC. Furthermore, tumor microenvironment and other genetic changes also have an important impact on the molecular characteristics and clinical outcomes of PTC, and need to be considered in PTC subtyping. In this regard, we broke the limitations of these mutation types in PTC, and used the NMF method [12, 13] to deconvolute the gene expression data of PTC and BTN samples so as to build new features of the PTC molecular landscape. Based on transcriptional landscape, PTC samples were classified as Immune-enriched, Stromal, BRAF-enriched and CNV-enriched subtypes; whilst BTN formed two subtypes. Higher frequency of distant metastasis and extrathyroidal extension were confirmed in the BRAF-enriched subtypes, which was supported by the previously reports [14, 15].

We identified a subgroup of PTCs with high immune cell infiltration and enhanced immune-related signaling, which was defined as Immune-enriched subtype. Its hub genes including LCP1, ACAP1, CCL19, CD3E, HLA-DRA, C1QA and CORO1A, were strongly associated with activities of immune cells. LCP1 was related to tumor infiltrating lymphocytes and plays an important role in lymphocyte formation and anti-cancer immune response [16]. The high expression of ACAP1 is positively associated with a variety of immune-related biological processes and pathways, such as adaptive immune response, T cell activation, and macrophage activation [17]. CCL19, as a candidate immunomodulator, regulates the adaptive immune response by increasing the interaction between dendritic cells, T cells and B cell [18]. As expected, patients with active immune profile were mostly complicated by Hashimoto’s thyroiditis and had a less aggressive pathologic characteristics. Moreover, through evaluating the specific patterns of immune cells infiltrating PTC, we found that the antitumor cells and cytokines were mainly found in Immune-enriched subtype. Whilst the protumor leukocytes were activated in Immune-enriched subtype, BRAF-enriched subtype and stromal subtype, which was supported by previous studies that different types of leukocytes play distinct role in PTC by releasing several cytokines [19, 20]. These findings collectively provided the molecular signature of immune-enriched subtype that may better explain the clinicopathological features of PTC with Hashimoto’s thyroiditis.

In addition, the gene expression of immune checkpoints (PD-1, PD-L1, CTLA-4) and T cells was upregulated in Immune-enriched subtype than other subtypes. Immune checkpoints were thought to be mainly related to self-tolerance [21], several studies indicated that the increased PD-L1 in cytotoxic T cells was induced by the IFN-γ during the anti-tumor immune reaction [22, 23]. A recent study reported that PTC patients with increased expression of immune checkpoint had a relatively better prognosis [22, 23], which supports our findings. Collectively, we identified a distinct Immune-enriched subtype of PTC with specific immune features and a better prognosis.

We also identified a BRAF-enriched Subtype of PTCs, which augmented with the activated the MAPK signaling, the ErbB signaling and the Notch signaling. BRAF-enriched Subtype had more extrathyroidal extension and was associated with advanced TNM stages compared to other types. BRAF^V600E^ mutation is the most common oncogenic mutation in PTC, which may correlates closely with aggressive tumor behavior and poor clinical outcomes of PTC [24]. BRAF is a serine-threonine kinase that can cause phosphorylation and activation of MAPK kinase and other downstream targets of the MAPK signaling pathway. Elevated MAP-kinase activity by BRAF^V600E^ mutations also have a strong positive effect on NOTCH downstream targets in thyroid cancer. We found that several genes, including LRRK2, SLC34A2, MUC1, and KRT19, played key regulatory roles in BRAF-enriched Subtype by targeting the oncogenic pathways. In our study, we confirmed that LRRK2 promote the proliferation of PTC through in vitro and in vivo studies. It was reported that the overexpression of SLC34A2, MUC1 and KRT 19 was significantly related to BRAF^V600E^ mutation and metastasis of PTC [25–27]. Recent studies show that the aggressiveness of BRAF^V600E^ mutation in PTC was impacted by various factors clinically, including age, sex, pathological subtypes, and other genetic alterations [28, 29]. Not all of the BRAF^V600E^ mutated PTC were at a high risk of recurrence [30]. However, the molecular characteristics of these highly aggressive BRAF-mutated PTCs is still unknown. Therefore, we performed additional analyses for the subclassification in BRAF-enriched Subtype and found that BRAF-enriched Subtype can be further divided into three subclasses. PTC with extrathyroidal extension were mainly concentrated in the BRAF-enriched-1, whilst the BRAF-enriched-2 showed increased immune infiltration and better clinicopathological features. Several oncogenes were centralized in the advanced clinical features of BRAF-enriched-1 Subtype, including CST6, NGEF, and ETV4 and BNIPL. Among them, the CST6 and ETV4 were respectively reported as downstream targets of BRAF mutation and involved in the regulation of the progression of PTC [31, 32]. NGEF was identified as a BRAF^V600E^-associated biomarker with prognostic value as potential BRAF^V600E^ inhibitor joint target [33]. In keeping with our finding, a single-cell RNA sequencing analysis also found the heterogeneity in PTC patients with BRAF mutations [8]. Collectively, our classification within the BRAF-enriched subtype would be helpful for clinical decision-making of BRAF-mutated PTC patients.

To determine the relationship of thyroid normal tissue, BTN and PTC, as well as dissect the evolutionary dynamics of four PTC subtypes, we performed the pseudotemporal trajectory analysis. The transcriptional profiles of BTNs were almost the same as those of BTN-adjacent thyroid tissues, which were located at the early developmental stage followed by PTCs belong to stromal subtype. With the accumulation of oncogenic factors and genes related to the immune microenvironment, PTC was divided into two branches as the immune-enriched subtype at lower branch and BRAF-enriched subtype at upper branch, further confirming the existence of the molecular classification of PTC. Currently, the ecological relationship between BTN and PTC remains unclear and controversial [34, 35]. Previous study suggested that BTN has independent origins different from PTC at the genomic level [34]. Our current study at least in part provides the evidence of the molecular links of the initiation and progression from BTN to subtypes of PTC.

Since distinguishing PTC from BTN is of great importance clinically, molecular diagnostic tests were helpful to improve the diagnostic performance and avoid patients from unnecessary thyroid surgery. In this regard, we built up a set of gene markers in our subtyping system for differential diagnosis. Twenty hub genes of these six differed subtypes could significantly differentially expressed between PTC and BTN with AUC up to 98.39% in discovery cohort and AUC of 90.04% at validation cohort. This indicates that the 20-gene expression signature has potential clinical applications in the differential diagnosis of PTC and BTN patients, which can be further verified in the fine needle aspiration biopsy samples in the following research. The most common used thyroid nodules classifiers, ThyroSeq v3 Genomic Classifier and Afirma Genomic Sequencing Classifier were consist of a large panel of genes and mainly used in the US [36]. Therefore, this 20-gene panels we proposed may be expected to provide a widely available solution for thyroid nodules differentiated diagnosis in Chinese population.

In conclusion, we demonstrated the transcriptional and genomic landscape of PTC and BTN. PTC could be classified as four molecular subtypes (Immune, Stromal, BRAF, and CNV-enriched) based on gene expression profiles that associated with clinicopathological features. Our integrative molecular analysis of the subtypes provides a new insight into the molecular features and links of the disease progression from BTN to PTC. We have also identified the novel gene-signature for distinguishing PTC from BTN.

## Methods

### Patients and samples

We retrospectively selected patients with thyroid surgery in the First Affiliated Hospital of Sun Yat-sen University or Sun Yat-sen University Cancer Center from 2011 to 2019, including 225 cases of PTC and 77 cases of BTN. When the thyroid tissue was excised in the operating room, the nodule and paramodular tissue were cut into particles with a length of 5 mm on the sterile curved disc. The calcified and necrotic areas were carefully removed. The isolated samples were placed in Eppendorf tubes and snap frozen in liquid nitrogen for the use of RNA-seq and other laboratory analyses. Postoperative pathology was used for diagnosis according to the criteria defined by World Health Organization. Clinical data, including demographic information and test results were collected retrospectively. Hashimoto’s thyroiditis was diagnosed according to postoperative pathology or when the serum TPOAb >35 IU/mL or TgAb >20 IU/mL. The detailed clinical information of patients was showed in Supplementary Table 1. Moreover, 497 PTC samples with genomic and transcriptomic profiles from The Cancer Genome Atlas (TCGA) were used for external validation. The study was conducted in accordance with the Declaration of Helsinki [37]. Informed consents were prospectively collected from all subjects that they were willing to participate in our project, and the study was approved by the Institutional Research Ethics Committee of the First Affiliated Hospital of Sun Yat-Sen University and Sun Yat-sen University Cancer Center.

### RNA extraction

About 60 mg of tissues were used for RNA isolation, which were ground into powder by liquid nitrogen and lysed in 1.0 mL TRIzol solution. Total RNA was extracted according to the manufacturer’s instructions. RNA integrity was assessed using the RNA Nano 6000 Assay Kit of the Bioanalyzer 2100 system (Agilent Technologies, CA, USA).

### Clustering and transcriptome sequencing

The clustering of the index-coded samples was performed on a cBot Cluster Generation System using TruSeq PE Cluster Kit v3-cBot-HS (Illumina) according to manufacturer’s instructions. After cluster generation, the library preparations were sequenced on an Illumina Novaseq platform, which were finished by Novogene Bioinformatics Institute. A total amount of 1 μg RNA per sample was used as input material for the RNA sample preparations. Briefly, mRNA was purified from total RNA using poly-T oligo-attached magnetic beads. Fragmentation was carried out using divalent cations under elevated temperature in First Strand Synthesis Reaction Buffer (5X). First-strand cDNA was synthesized using random hexamer primer and M-MuLV Reverse Transcriptase. Second-strand cDNA synthesis was subsequently performed using DNA Polymerase I and RNase H. Remaining overhangs were converted into blunt ends via exonuclease/polymerase activities. After adenylation of 3’ ends of DNA fragments, Adaptor with hairpin loop structure were ligated to prepare for hybridization. In order to select cDNA fragments of preferentially 370~420 bp in length, the library fragments were purified with AMPure XP system (Beckman Coulter, Beverly, USA). Then PCR was performed with Phusion High-Fidelity DNA polymerase, Universal PCR primers and Index (X) Primer. At last, PCR products were purified (AMPure XP system) and library quality was assessed on the Agilent Bioanalyzer 2100 system.

### Quality control

Raw data (raw reads) of fastq format were firstly processed through in-house per l scripts. In this step, clean data (clean reads) were obtained by removing reads containing adapter, reads containing ploy-N and low-quality reads from raw data. At the same time, Q20, Q30 and GC content were calculated. All the downstream analyses were based on the clean data of high quality. After quality control, we finally included 216 cases of PTC and 75 cases of BTN into the subsequent analysis.

### Quantification of gene expression level

Human reference genome (hg19) and gene model annotation files were downloaded from UCSC. Index of the reference genome was built and paired-end clean reads were aligned to the reference genome using HISAT2 with default parameters. RSeQC was used to measure gene expression abundance as reads per kilobase per million mapped reads (RPKM).

### Fusion analysis

We used STAR-Fusion v1.9.1 to detect genes that are fused, require at least 5 split reads and 2 read-pairs spanning the fusion event to filter out low confident fusion event. To reduce the false positive fusion events, we removed any fusions where expression of both genes in the gene pair was found to be RPKM (Reads per kilo base per million mapped reads) value less than 1 across all samples. To remove spurious fusions, we filtered all fusions which gene pair distance was less than 10Kb that annotated as read-through event.

### Mutation detection

Somatic mutations were detected by MuTect based on alignment file and high confident somatic mutations were met defined as follows: (I) both the tumor and normal samples should be covered sufficiently (≥10×) at the genomic position; (II) the variants should be supported by at least 5% of the total reads in the tumor while less than 1% in the normal; (III) the variants should be supported by at least five reads in the tumor. All high confident somatic mutations were filtered out by the dbSNP (version 135) site which is commonly polymorphic without known medical impact. The remaining mutations were annotated with ANNOVAR and subjected to subsequent analyses.

### Copy number variation analysis

We used CaSpER [38] to identify and visualize copy number variations (CNV), which is a signal processing tool that uses RNA-seq information to detect focal and large-scale CNV events in multiscale resolution. B-allele frequencies were generated from RNA-seq data to decipher allelic imbalances in sample. CNVs were called based on expression level and B-allele frequencies information.

### Identification of molecular subtypes

To perform unsupervised clustering, a series of genes were filtered using the following criteria: (1) genes expressed at low levels (RPKM < 1.0) in more than half of samples, and (2) genes of low variance across samples (coefficient of variation <0.8). The remaining 1585 genes were log-transformed and used for subsequent unsupervised clustering. The unsupervised consensus NMF was performed on the selected gene-by-sample matrix with the number of factors set to 6. Once the mRNA expression matrix was deconvoluted by NMF, we listed a set of distinct exemplar genes for the *i*th factor by ranking genes in descending order of the difference between the loading value in the *i*th factor and the largest loading value not in the *i*th factor of the loading matrix. To further characterize the molecular functions of each subtype, we then calculated the expression fold-change of each gene between a specific subtype and other subtypes. Genes were then sorted in descending order according to their expression fold-change, and GSEA analysis in pre-rank mode was performed to infer enriched pathways for each subtype.

To identify gene markers that can distinguish different molecular subtypes, random forest models were trained for each subtype. For each subtype, we first classified the whole patients into two group, that is patients classified as the given subtype or patients that are not classified as the given subtype. Using the previous exemplar genes as feature vector, random forest model was established and the importance of each inputted genes was evaluated. Using a feature permutation test [39], we selected genes with adjusted *P*-value lower than 0.05 as significant markers for each molecular subtype.

### Validation in TCGA database

The reliability of our molecular subtype was further validated in the thyroid cancer cohort from TCGA project. We downloaded the mRNA raw sequencing data from GDC data portal, and performed quantification analysis followed the previous mentioned procedure. Using the derived marker genes of each molecular subtype, the Nearest Template Prediction (NTP) algorithm was applied to classify the TCGA samples into corresponding molecular subtype.

### Calculation of thyroid differentiation score (TDS)

The thyroid differentiation score (TDS) was calculated to quantify relationships between thyroid differentiation and diverse genetic or epigenetic events. 16 thyroid function genes were selected to calculate the TDS score according to previous literatures [6]. For each of the 16 selected genes, we first calculated the median value of log_2_ RPKM across samples. Then, the expression level of the 16 thyroid function genes were median centered, and summed up to produce the TDS score in each sample.

### Immune score and stromal score

To quantify the activity of immune system, an immune-related gene set that comprised 66 immune cell surface markers and immune regulation genes were collected from published literatures [40, 41]. The gene set variation analysis (GSVA) was applied on each sample based on the collected immune-related gene set. The GSVA score was taken as an indicator for quantifying the activity of immune system, and defined as immune score. A higher immune score corresponded to more active immunological effects. Using the ABSOLUTE [42] software, we also calculated the stromal score for each sample.

### Hub genes analysis

To identify hub genes that highly correlated with marker genes in each molecular subtype, an analysis pipeline was performed as below. We first constructed a transcriptional interactions network using the ARACNe (Algorithm for Reconstruction of Accurate Cellular Networks) method [43] in our patient cohort. The previously identified subtype marker genes were treated as hubs in the ARACNE mutual information (MI) calculation. Adaptive Partitioning was then applied to find downstream co-expression genes of the inputted hub markers. The final constructed network was subsequently inputted to Cytoscape [44]. Based on the transcriptional interactions network, we used the Maximal Clique Centrality (MCC) algorithm in cytoHubba [45] to identify top hub genes in the network. Given these top hub genes, the first-layer neighbors were extracted from the transcriptional interactions network and visualized by Cytoscape. To reveal the downstream regulated pathway of the identified hub genes, we performed GSEA analysis for their downstream regulated genes. Specifically, the downstream regulated genes were first ranked by their fold-change of expression values in the corresponding subtype relative to other subtypes. Then, enriched pathways were identified using the pre-rank module in GSEA package. The interactions between hub genes and downstream regulated pathways were quantitatively defined by their average normalized enrichment score, and visualized by Cytoscape.

### Inference of the pseudotemporal trajectory for benign and malignant thyroid nodules

To uncover the potential molecular developmental process of thyroid nodules from benign status to malignant status, a pseudotemporal trajectory analysis were performed in our study. By assuming that each collected patient may have different progression of disease, the analysis was performed based on each sequencing sample. Using the highly variable expression genes selected from previous step, we constructed a transcriptional profile for each sample. Monocle2 [46] was adopted to infer pseudotemporal trajectory in our cohort using BTN-adjacent samples as starting point (which considered as normal thyroid tissue). A Reversed Graph Embedding algorithm was performed to reduce the data’s dimensionality. With the expression data projected into a lower dimensional space, inputted samples were ordered in pseudotime and trajectory was built to describe how patient samples transit from one state into another. To identify genes that were differentially expressed in the decision point of pseudotemporal trajectory, branched expression analysis modeling (BEAM) was performed. By filtering with FDR *q*-value <0.05, a series of fate-decision genes were found and visualized in an expression heatmap. Besides, to reveal the progression status for each pseudotime, the molecular subtypes, TDS, immune enrichment score and BRAF mutation status were manually mapped and visualized in the trajectory.

### Identification of potential markers for malignant thyroid nodules diagnosis

In order to identify potential markers for distinguishing malignant thyroid nodules from benign nodules, we performed an analysis approach using hub genes identified from the previously constructed molecular subtypes. From all available samples, 80% were used for the training set, and the remaining 20% were randomly selected for the external test set. Notably, to maintain the original distribution, the ratio of benign nodules to malignant nodules was kept the same as in the original data set for both training and test set. Using the training data set, we first constructed a classification model by the Logistic Regression (LR) algorithm. Recursive feature elimination (RFE) was then applied to find the optimal number of marker genes. By recursively eliminating a small number of genes per loop, RFE attempts to eliminate dependencies and collinearity that may exist in the model. We used cross-validation to score different gene subsets and select the best scoring collection of genes during the recursive procedure. After finding the optimal subset of markers, 4,6,8,10-fold cross-validation was performed on the training set to evaluate the prediction performance, and the Receiver Operator Characteristic (ROC) curves were drawn. Also, using the external test set, we assessed the robustness of our constructed model by computing the area under ROC curve (AUC). Finally, the expression level of the selected panel was visualized in a heatmap, and the fold change (FC) between malignant nodules and benign nodules was also presented. To further validate the stability of our selected marker genes, prediction models for distinguishing benign nodules and malignant nodules were also constructed using Support Vector Machine (SVM), Naïve Bayes (NB), Random Forest (RF) and K-Nearest Neighbor (KNN) algorithm. The performances in training set and independent test set were evaluated as described above.

### Immunohistochemistry (IHC) and fluorescent multiplexed immunohistochemistry (mIHC)

Paraffin-embedded thyroid nodule tissues were sliced into 4 um thickness. After deparaffinization, the slides were blocked with 20% goat serum. The sections were then incubated with primary antibodies of SLC34A2 (Cell Signaling Technology) and LRRK2 (Cell Signaling Technology) at 4 °C overnight followed by secondary antibody incubation. For mIHC, the slides were then nurtured in a secondary horseradish peroxidase-conjugated polymer to induce the binding of different fluorophores via tyramide signal amplification (Panovue). The protein expression of CD163, CD86, CD8, Foxp3 were evaluated.

### Quantitative real-time PCR (qRT-PCR)

cDNA was synthesized by reverse transcription from total RNA using a PrimeScript^TM^ RT-PCR Kit (TaKaRa, CA). qRT-PCR was then performed using SYBR Premix Ex Taq^TM^ (TaKaRa, CA) on a LightCycler 480 system. GAPDH was used as an internal control to normalize the expression levels of mRNA. Relative mRNA expression were analyzed by the 2^—ΔΔCT^ method.

### Western blot analysis

Protein was extracted by RIPA lysis buffer with Protease and Phosphatase Inhibitor Cocktail, and protein concentration were measured by the BCA Protein Assay Kit (ZJ102, Epizyme). Proteins of each sample was separated by sodium dodecyl sulfate polyacrylamide gel and transferred onto PVDF membranes. After blocking by 5% skim milk in TBS-T at room temperature, the membranes were incubated with primary antibody at 4 °C overnight, including LRRK2 (91882, Cell Signaling Technology) and GAPDH (as a reference, HRP-60004, Proteintech). The membranes were incubated with HRP-linked secondary antibody and detected by chemiluminescence.

### Small interfering RNA (siRNA), short hairpin RNA (shRNA) transfection

The siRNA and shRNA targeting LRRK2 and negative control were purchased from GenePharma (Shanghai, China) and Genechem (Shanghai, China) respectively. Thyroid cancer cells were seeded in 6-wells plates and cultured for 24 h, and then tranfected with siRNA or lentivirus with shRNA using Lipo3000 Transfection Kit (Invitrogen). After transfection, the cells were harvested for further experiments.

### Cell culture and proliferation assays

The thyroid cancer cell lines BCPAP and KHM-5M were purchased from the Cell Culture Collection of the Chinese Academy of Sciences (Shanghai, China), and both were cultured in RPMI-1640 (Gibco) with 10% fetal bovine serum (Gibco). Cells were cultivated at 37 °C with 5% CO_2_. For colony formation assay, cells were seeded in 6-wells plate for 2 weeks. Colonies were then fixed with 4% paraformaldehyde for 15 min and stained with crystal violet for 10 min. For cell proliferation assay, cells were plated in 96-well plates, and incubated with Cell Counting kit-8 assay (CCK8, Dojindo Laboratories) for 2 h. We recorded the absorbance of each well with a Multimode Microplate Reader (Thermo).

### Xenograft tumor model in mice

To establish xenograft tumor models, KHM-5M (5 × 10^6^/mouse) with stable LRRK2-knockdown or negative control were subcutaneously injected into the right flank of 4-weeks old female BALB/c nude mice. All mice were randomized into control and experimental groups. Tumor sizes were measured every 3 days and calculated according to the formula: length × width^2^ × 0.5. Tumor were token photos and weighted after sacrifice of mice. Animal studies were approved by the Animal Experimentation Ethical Committee of The First Affiliated Hospital of Sun Yat-sen University.

### Statistical analysis

All data analyses were conducted in R 4.0.3. The comparison of immune enrichment scores, expression levels of immune check point genes and GEP score were performed using Wilcoxon rank sum test. The correlation analysis between clinical features and molecular subtypes was conducted with Chi-square independence test. Statistical significance was defined as a two-sided *P* value of less than 0.05.

## Supplementary information

Supplementary table 1

Supplementary figures

## Data Availability

The raw RNA-sequencing data generated in this study will be deposited in approved database with accession number at the time of publication. The TCGA THCA dataset were used in this study. Additional data related to this article will be shared on reasonable request to the corresponding author.

## Code availability

The custom computer code will be shared on reasonable request to the corresponding author.
