# Supplementary material for: Distinct molecular subtypes of papillary thyroid carcinoma and gene signature with diagnostic capability
Source: Oncogene. 2022 Oct 17;41(47):5121–32. doi: 10.1038/s41388-022-02499-0 (PMC9674518; doi:10.1038/s41388-022-02499-0)
Supplement: Supplementary file 1 — Supplementary table 1 [file 41388_2022_2499_MOESM1_ESM.docx]

| **Supplementary Table 1.** Clinicopathologic comparison of 4 molecular subtypes of PTC in training cohort. | | | | | |
| --- | --- | --- | --- | --- | --- |
| **Clinical features** | **Subtype 2** | **Subtype 3** | **Subtype 4** | **Subtype 6** | ***P* value** |
| Sex |  |  |  |  | 0.092 |
| Female | 32 (84.2%) | 19 (61.3%) | 70 (79.5%) | 20 (69.0%) |  |
| Male | 6 (15.8%) | 12 (38.7%) | 18 (20.5%) | 9 (31.0%) |  |
| Age (yrs) | 38.68 ± 12.00 | 37.82 ± 11.03 | 41.44 ± 13.01 | 39.68 ± 9.975 | 0.403 |
| Multifocality | 11 (28.9%) | 8 (25.8%) | 36 (40.4%) | 8 (27.6%) | 0.288 |
| Extrathyroidal extension | 3 (7.9%) | 6 (19.4%) | 25 (28.1%) | 2 (6.9%) | **0.013** |
| PTMC | 21 (55.3%) | 22 (71.0%) | 26 (29.9%) | 11 (37.9%) | **< 0.001** |
| Tumor size |  |  |  |  | **0.002** |
| ≤ 2 cm | 36 (94.7%) | 28 (90.3%) | 63 (71.6%) | 27 (93.1%) |  |
| > 2 cm | 2 (5.3%) | 3 (9.7%) | 25 (28.4%) | 2 (6.9%) |  |
| Lymph node metastasis | 20 (52.6%) | 16 (51.6%) | 57 (64.0%) | 18 (62.1%) | 0.450 |
| Distant metastasis | 0 (0.0%) | 2 (6.5%) | 3 (3.4%) | 1 (3.6%) | 0.511 |
| Stage |  |  |  |  | **0.043** |
| Ⅰ + Ⅱ | 38 (100.0%) | 29 (93.5%) | 79 (88.8%) | 29 (100.0%) |  |
| Ⅲ + Ⅳ | 0 (0.0%) | 2 (6.5%) | 10 (11.2%) | 0 (0.0%) |  |
| Note: Age was presented as mean ± SD. | | | | | |
| Abbreviations: PTMC, Papillary Thyroid Microcarcinoma. | | | | | |
