# Supplementary material for: Distinct molecular subtypes of papillary thyroid carcinoma and gene signature with diagnostic capability
Source: Oncogene. 2022 Oct 17;41(47):5121–32. doi: 10.1038/s41388-022-02499-0 (PMC9674518; doi:10.1038/s41388-022-02499-0)
Supplement: Supplementary file 2 — Supplementary figures [file 41388_2022_2499_MOESM2_ESM.docx]

**Supplementary Figure 1. The genomic landscape of thyroid nodules. (a)** Mutation density across the cohort. **(b)** The frequency of somatic copy number variations (CNVs) across 22 chromosomes, with gains in red and losses in blue. **(c)** Number and frequency of recurrent mutations, fusion events and CNV across the cohort. **(d)** RNA expression fusion plots of RET, NTRK3, NTRK1, ALK and PPARG fusions were drawn from 5’ to 3’. The pairs of numbers below indicate the paired-end supporting reads from RNA-seq. The number of samples detected and split reads were indicated on the upper right and left, respectively. **(e)**The KCTD5-RET fusion was detected by RT-PCR and agarose gel electrophoresis in target sample (1075T) and its paired para-tumor sample (1075P) and other three pairs of PTC and para-tumor samples. **(f)**Bar graph represented the relative expression of KCTD-RET detected by Sanger sequencing.


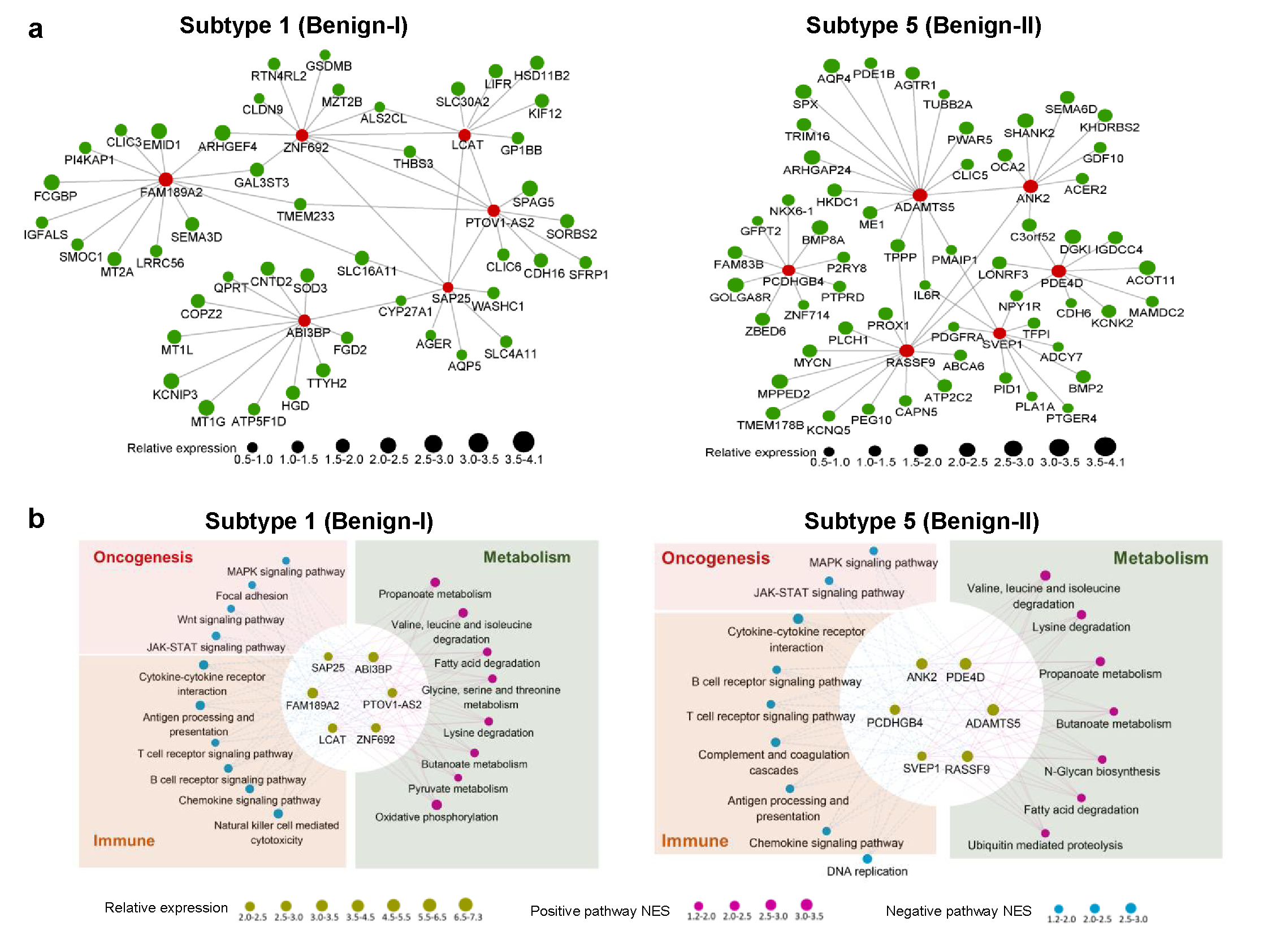


**Supplementary Figure 2. Gene expression characteristics of BTN subtypes.** **(a)** Gene correlation network represents hub genes of Subtype 1(Benign-I) and Subtype 5(Benign-II) identified by ARACNe method. **(b)** The gene-pathway co-expression network of Subtype 1(Benign-I) and Subtype 5(Benign-II) by the ARACNe and GSEA method. Dots in the center of the network stand for top hub genes, and the size of dots represents the relative expression level of each gene. Red dots and blue dots in the outer circle stand for the downstream upregulated and downregulated pathways of the hub genes, and dots’ size represents normalized enrichment score (NES).


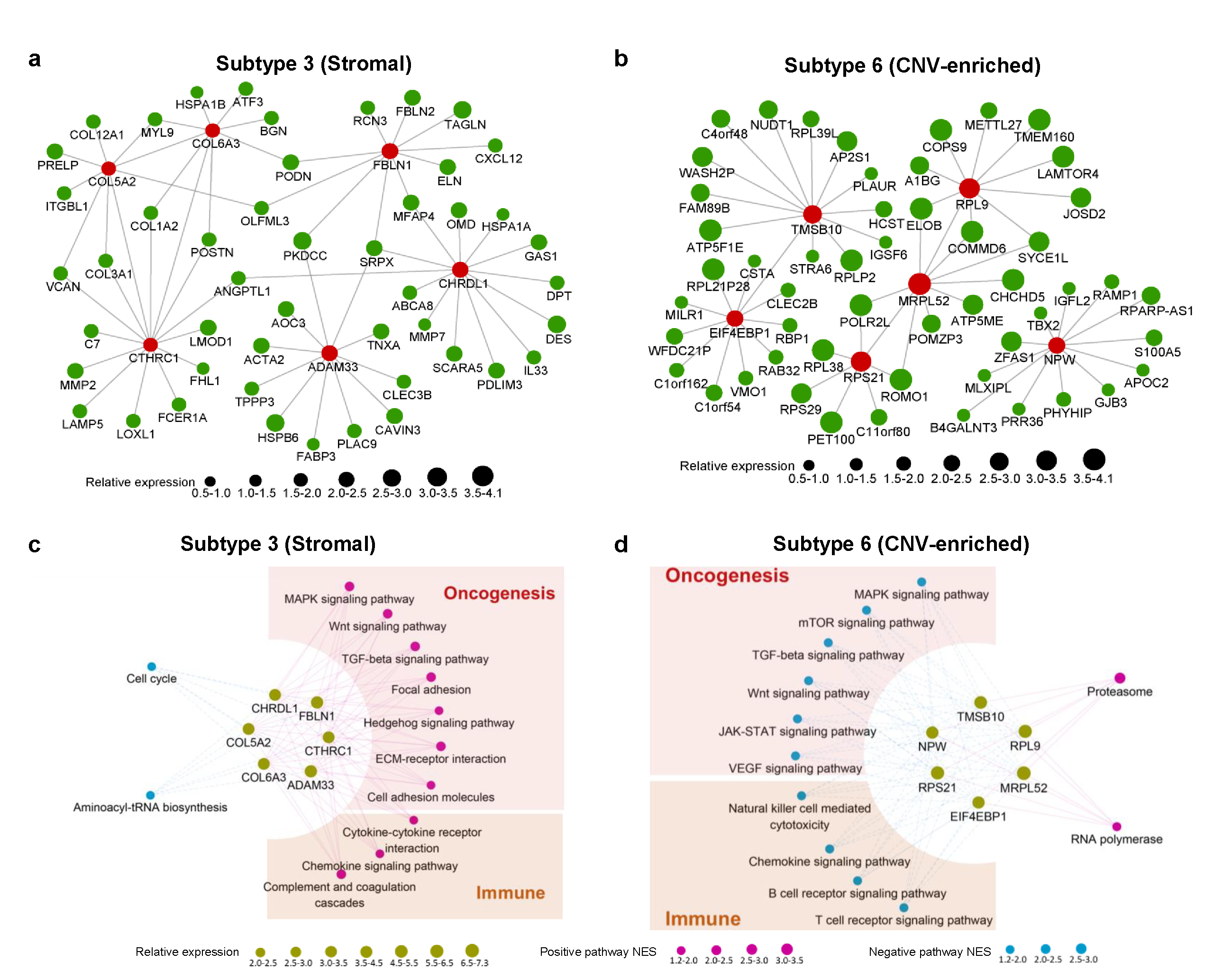


**Supplementary Figure 3. Gene expression characteristics of Stromal subtype and CNV-enriched subtype.** Gene correlation network represents hub genes of Stromal Subtype **(a)** and CNV-enriched Subtype **(b)** identified by ARACNe method. The gene-pathway co-expression network of Stromal Subtype **(c)** and CNV-enriched Subtype **(d)** by the ARACNe and GSEA method. Dots in the center of the network stand for top hub genes, and the size of dots represents the relative expression level of each gene. Red dots and blue dots in the outer circle stand for the downstream upregulated and down regulated pathways of the hub genes, and dots’ size represents normalized enrichment score (NES).


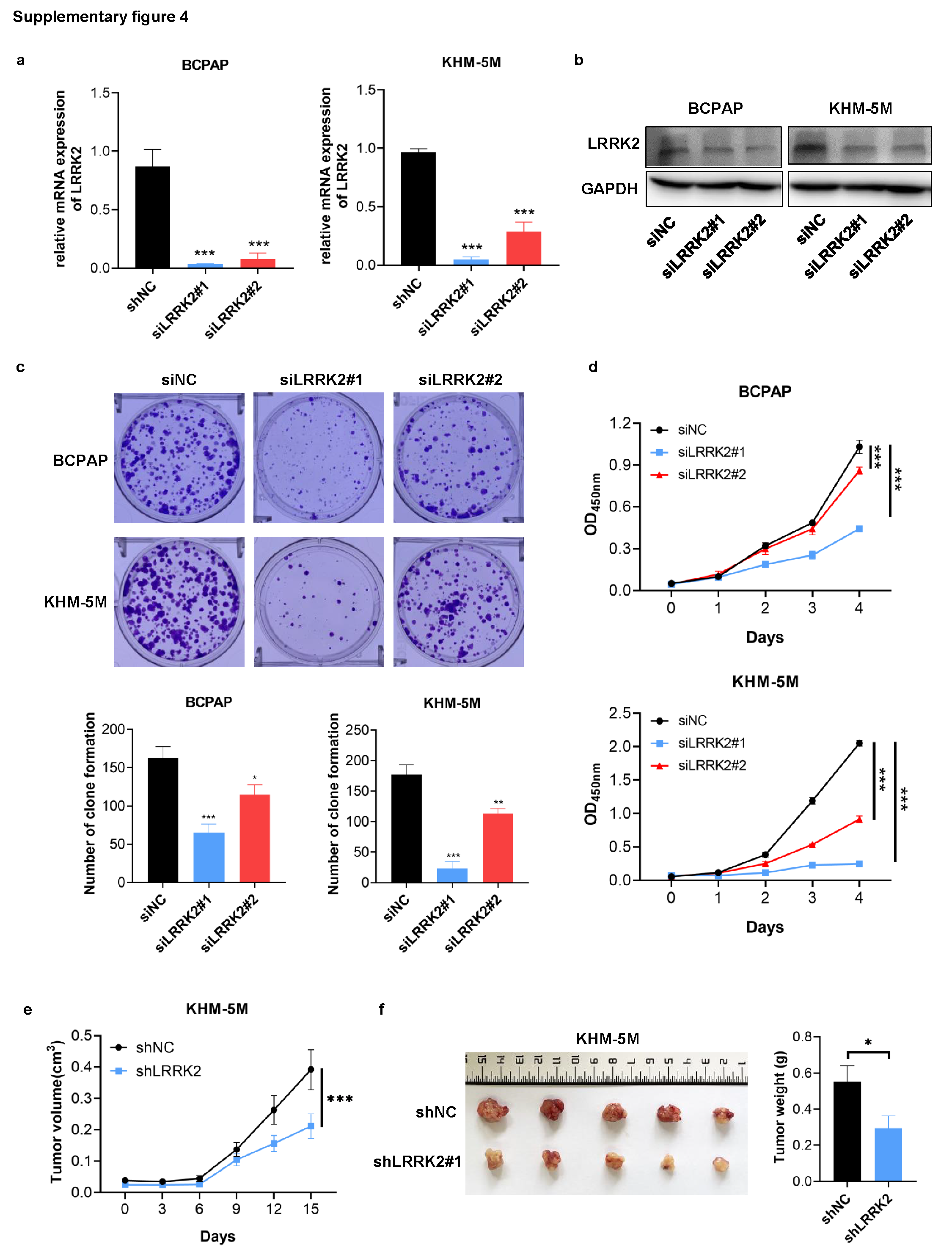


**Supplementary Figure 4. LRRK2 promote proliferation of thyroid cancer in vitro and in vivo.** **(a)** qRT-PCR and **(b)** Western blot showed the knockdown efficacy of LRRK2 in BCPAP and KHM-5M. **(c)** Colony formation and (d) CCK8 assay were used to explore the proliferation of LRRK2 knockdown cells. **(e)** Growth curve of subcutaneous tumors in KHM-5M injected mice. **(f)** Images of subcutaneous tumors of LRRK2 knockdown group and control group. Weight of subcutaneous tumors were quantified.


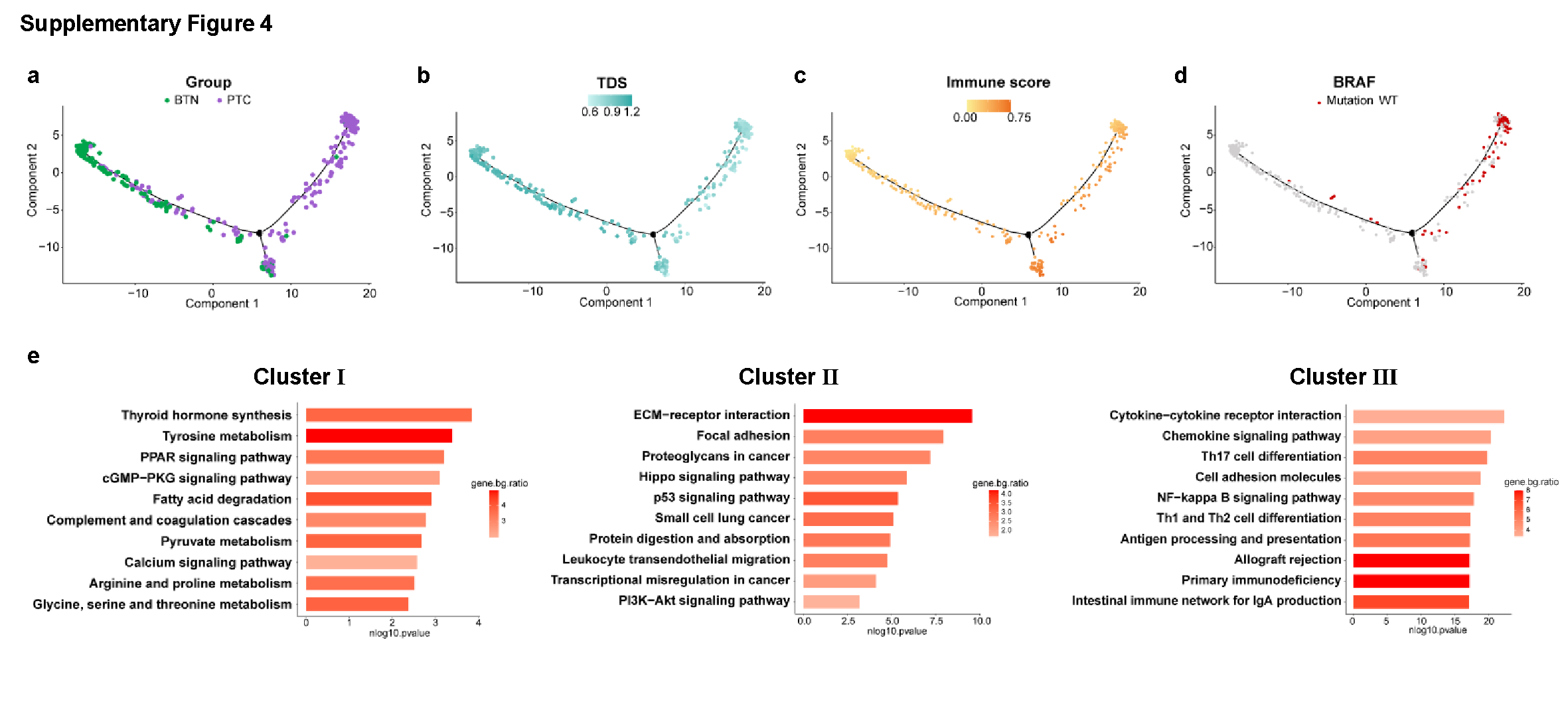


**Supplementary Figure 5. Gene expression dynamics of thyroid nodules. (a)** Pseudotime trajectory reconstruction of RNA -seq profiles of all thyroid nodules using Monocle 2. Each point represents a patient and is color-coded by BTN (green) and PTC (purple). Pseudotime trajectory of RNA-seq profiles of thyroid nodules, and colored by calculated TDS using the expression of 16 thyroid function genes **(b)** calculated immune score using the expression of immune exemplar genes **(c)** and BRAF status **(d)**. **(e)** The top 10 changed pathways of each gene cluster by KEGG pathway enrichment analysis.
